# Coronary artery calcium and multiparametric cardiovascular risk assessment in non-dialysis chronic kidney disease

**DOI:** 10.17305/bb.2026.14105

**Published:** 2026-06-18

**Authors:** Ivana Đuran, Nikola Komazec, Bojana Ljubičić, Ana Lazarević, Siniša Živković, Tijana Azaševac, Sonja Golubović, Mia Manojlović

**Affiliations:** 1 Faculty of Medicine in Novi Sad, University of Novi Sad, Novi Sad, Serbia; 2 Clinic of Nephrology and Clinical Immunology, University Clinical Center of Vojvodina, Novi Sad, Serbia; 3 Institute of Cardiovascular Diseases of Vojvodina, Sremska Kamenica, Serbia; 4 Department of Emergency Internal Medicine, Emergency Center, University Clinical Center of Vojvodina, Novi Sad, Serbia; 5 Clinic for Endocrinology, Diabetes and Metabolic Disorders, University Clinical Center of Vojvodina, Novi Sad, Serbia

**Keywords:** Chronic kidney disease, coronary artery calcium score, Agatston score, cardiovascular risk, CT angiography, vascular calcification, ventricular dysfunction

## Abstract

Cardiovascular disease is a leading cause of mortality in chronic kidney disease (CKD), but whether impaired renal function independently increases coronary artery calcium (CAC) burden in patients with comparable cardiovascular referral indications remains uncertain. This retrospective cross-sectional study included 206 adults who underwent coronary computed tomography angiography (CCTA) with simultaneous Agatston scoring at the Institute for Cardiovascular Diseases of Vojvodina between 2020 and 2025. Patients were classified as having eGFR-defined non-dialysis CKD stages 3–5 (*n* ═ 110; estimated glomerular filtration rate [eGFR] ≤ 59 mL/min/1.73 m^2^) or preserved renal function (*n* ═ 96; eGFR ≥ 60 mL/min/1.73 m^2^). Both groups were drawn from the same cardiovascular referral population. The primary outcome was total Agatston score, while secondary analyses included Agatston severity categories, significant vessel-level stenosis, calcified coronary segment count, eGFR–Agatston correlation, and predictors of severe CAC. Total Agatston scores did not differ significantly between CKD and control groups (median 248.5 vs. 198.0; *P ═* 0.240), and severe calcification was similarly frequent (40.0% vs. 41.6%). Vessel-level stenosis, calcified segment distribution, and the eGFR–Agatston correlation were also non-significant. In multivariable logistic regression adjusted for age, sex, hypertension, diabetes, and prior coronary artery bypass grafting (CABG), CKD was not independently associated with severe CAC. However, CKD patients showed a markedly more adverse cardiovascular phenotype, including more hypertension, angina, peripheral arterial disease, prior CABG, lower left ventricular ejection fraction (LVEF), higher right ventricular systolic pressure (RVSP), and more severe valvular disease. These findings suggest that CAC scoring alone does not capture the multidimensional cardiovascular burden of non-dialysis CKD and should be integrated with echocardiographic and systemic vascular assessment in high-risk referral populations.

## Introduction

Chronic kidney disease (CKD) affects an estimated 850 million individuals globally and is the most common non-communicable condition contributing to premature cardiovascular mortality [1]. Cardiovascular disease accounts for approximately half of all deaths in CKD patients, with the excess risk progressively increasing as the glomerular filtration rate (GFR) declines, independent of conventional risk factors such as diabetes mellitus and arterial hypertension [1, 2]. This disproportionate cardiovascular burden reflects not only the high prevalence of shared risk factors—including hypertension, dyslipidemia, and diabetes—but also a constellation of CKD-specific mechanisms that collectively accelerate cardiovascular damage. These mechanisms include chronic systemic inflammation, oxidative stress, endothelial dysfunction, secondary hyperparathyroidism, hyperphosphatemia, uremic toxin accumulation, and elevated fibroblast growth factor 23 (FGF-23), all of which independently contribute to atherosclerosis progression and myocardial injury [1, 3, 4]. As kidney function deteriorates, a parallel accumulation of structural cardiac changes occurs: left ventricular hypertrophy (LVH), systolic and diastolic dysfunction, valvular calcification, and pulmonary hypertension collectively constitute what is now recognized as CKD-associated cardiomyopathy [2, 5].

Among the cardiovascular complications of CKD, vascular calcification holds a particularly central position. Its prevalence rises markedly with advancing renal impairment, ranging from 40%–60% in CKD stages 3–5 to 80%–90% in end-stage kidney disease patients on dialysis [6]. Two mechanistically and prognostically distinct processes underlie coronary calcification in this population. Intimal atherosclerotic calcification develops within atherosclerotic plaques through oxidized lipid deposition, macrophage infiltration, and foam cell formation—a pathway that closely parallels the mechanism in the general population and reflects overall atherosclerotic burden. Medial (Mönckeberg) sclerosis, by contrast, is a non-atherosclerotic process characterized by the transdifferentiation of vascular smooth muscle cells into osteoblast-like phenotypes, driven by hyperphosphatemia, FGF-23 excess, Klotho deficiency, and disrupted calcium-phosphate homeostasis [3, 4, 7]. This latter mechanism becomes increasingly prevalent as CKD progresses, contributing to arterial stiffness, impaired coronary perfusion, and augmented left ventricular afterload independently of luminal atherosclerosis [7, 8]. A critical analytical limitation is that standard non-contrast computed tomography (CT) calcium scoring cannot distinguish between these two processes: it quantifies total calcium area and density without identifying the anatomical compartment involved, which has direct implications for the interpretation of calcium scores in CKD patients [5, 7].

The coronary artery calcium score (CACS), obtained by non-contrast multidetector CT using the Agatston method, is a validated, widely used measure of coronary atherosclerotic plaque burden and an established independent predictor of future major cardiovascular events in the general population [8]. In CKD, CACS has demonstrated incremental predictive value over conventional risk scores, with higher calcium loads correlating with greater cardiovascular event rates and all-cause mortality [6, 9]. Nevertheless, its interpretation in the context of CKD is considerably more complex. The coexistence of intimal and medial calcification mechanisms, compounded by a substantially elevated baseline cardiovascular comorbidity burden, confounds direct comparison with general population norms. Moreover, a critical methodological concern is that most studies comparing CACS in CKD vs non-CKD patients have recruited controls from the general population or from low-risk outpatient settings, where the baseline atherosclerotic burden is substantially lower [6, 10]. This design choice may lead to an overestimation of the independent contribution of impaired renal function to coronary calcium accumulation, as any difference in CACS between groups may reflect differential comorbidity burden rather than the isolated effect of reduced GFR.

Whether reduced kidney function per se drives a greater coronary calcium burden when CKD patients are compared against a non-CKD population with a similarly high cardiovascular risk profile remains an open and clinically consequential question. If the excess calcium burden observed in CKD studies largely reflects shared comorbidities rather than kidney disease itself, then calcium scoring thresholds and risk stratification tools calibrated in general population cohorts may require reappraisal before application in CKD-specific cardiovascular settings. To address this knowledge gap, we conducted a retrospective cross-sectional study at a tertiary cardiovascular referral center—the Institute of Cardiovascular Diseases of Vojvodina, Sremska Kamenica—from 2020 to 2025. The study design deliberately recruited both CKD patients and controls from the same cardiovascular referral population sharing broadly similar coronary computed tomography angiography (CCTA) referral indications, thereby reducing—but not eliminating—confounding from differential comorbidity burden. The primary endpoint was the between-group difference in total Agatston score. Secondary endpoints included Agatston severity categories, vessel-level stenosis ≥ 50%, number of calcified coronary segments, the correlation between eGFR and total Agatston score, and adjusted analysis of severe coronary calcification (Agatston >400) [10].

**Table 1:** Baseline demographic and clinical characteristics of patients with and without CKD

		**CKD group**	**Non-CKD group**	**HL estimate** **[95% CI]**	***P***
Age	Median (IQR)	71 (9.0)	67 (12.2)	−4.0 [−6.0, −2.0]	0.002^a^
BMI	Median (IQR)	26.1 (5.1)	27.2 (5.4)	0.7 [−0.3, 1.8]	0.181^a^
Sex, male	*n* (%)	68 (61.8%)	59 (61.5%)	/	0.958^b^
Smoker	*n* (%)	36 (32.7%)	37 (38.5%)	/	0.384^b^
SBP	Median (IQR)	140 (38.7)	130 (30.0)	−10.0 [−15.0, −0.01]	0.032^a^
DBP	Median (IQR)	80 (20.0)	80 (20.0)	−0.01 [−5, 0.01]	0.296^a^

^a^Mann-Whitney U test, ^b^chi-square test. **Abbreviations**: BMI, body mass index; CI, confidence interval; CKD, chronic kidney disease; DBP, diastolic blood pressure; HL, Hodges–Lehmann estimator; IQR, interquartile range; *n*, number; SBP, systolic blood pressure.

## Materials and methods

### Study design and population

This retrospective cross-sectional study was conducted at the Institute for Cardiovascular Diseases of Vojvodina (IKVB), Sremska Kamenica, Serbia. Data were collected from consecutive patients who underwent CCTA with simultaneous measurement of the Agatston score between January 2020 and December 2025. A total of 206 patients with complete clinical, laboratory, and echocardiographic data were included. All patients were referred for CCTA based on clinical suspicion of, or an established diagnosis of, coronary artery disease (CAD), ensuring that both study groups shared the same imaging indications and were drawn from the same cardiovascular referral population, although substantial differences in cardiovascular comorbidity burden between groups were nonetheless present. The difference in group sizes (CKD *n* ═ 110 vs. controls *n* ═ 96) was not coincidental: the CKD group comprised all consecutive patients satisfying the inclusion criteria over the study period, while the control group was sized to achieve an approximately 1:1 ratio through random sampling, with the final number determined by the available pool of eligible controls after applying exclusion criteria.

Patients were classified into two groups based on estimated glomerular filtration rate (eGFR) calculated using the Chronic Kidney Disease Epidemiology Collaboration (CKD-EPI) equation from a plasma creatinine value obtained at the time of CT imaging. The CKD group (*n* ═ 110) comprised patients with CKD stages 3–5 (eGFR ≤ 59 mL/min/1.73 m^2^), enrolled consecutively over the study period; all patients who satisfied the inclusion criteria were included without further selection. The control group (*n* ═ 96) comprised patients with normal or mildly reduced renal function (eGFR ≥ 60 mL/min/1.73 m^2^), randomly selected from the institutional CCTA database using a computer-generated random number sequence during the same period. Controls were selected without stratification by imaging indication or calendar period; all consecutively registered patients with eGFR ≥ 60 mL/min/1.73 m^2^ during the study period were eligible, and the random number sequence was applied to this pool without further restriction. Exclusion criteria, applied uniformly to both groups, comprised: incomplete medical documentation, missing CCTA or Agatston score data, absence of essential laboratory parameters, acute kidney injury at the time of evaluation, and age below 18 years. The institutional CCTA database contained approximately 8,000 examinations performed during the study period. Patients were not screened sequentially from this pool: the CKD group was enrolled consecutively among all patients satisfying the inclusion criteria, while controls were randomly selected from the database using a computer-generated sequence. The study flow and group allocation are summarized in Figure 1. A formal count of sequentially screened and excluded patients is therefore not available, and a Consolidated Standards of Reporting Trials (CONSORT)-style flow diagram was not feasible for this study.

### Clinical definitions

CKD was defined in accordance with the 2012 Kidney Disease: Improving Global Outcomes (KDIGO) Clinical Practice Guidelines as kidney damage or eGFR <60 mL/min/1.73 m^2^ persisting for ≥ 3 months [11]. Staging followed the KDIGO classification: stage 3 (eGFR 30–59 mL/min/1.73 m^2^), stage 4 (eGFR 15–29 mL/min/1.73 m^2^), and stage 5 (eGFR <15 mL/min/1.73 m^2^, excluding patients receiving chronic hemodialysis). eGFR was derived from a single plasma creatinine measurement using the 2021 CKD-EPI creatinine equation, which does not include a race coefficient [12]. For the majority of patients in the CKD group, reduced kidney function was an established diagnosis documented in the institutional medical record prior to CT imaging, supported by prior nephrology evaluations and consistent prior laboratory results. The single creatinine measurement obtained at the time of CT was used for formal group assignment and was consistent with the pre-existing clinical diagnosis in these cases. Nevertheless, strict KDIGO chronicity confirmation was not uniformly retrievable, and the term ‘CKD stages 3–5’ should be understood as eGFR-based stratification rather than formally confirmed CKD throughout this manuscript. Arterial hypertension was defined as systolic blood pressure ≥ 140 mmHg, diastolic blood pressure ≥ 90 mmHg, or current use of antihypertensive therapy. Diabetes mellitus was defined as fasting plasma glucose >6.6 mmol/L and/or use of oral antihyperglycemic agents or insulin. Dyslipidemia was defined as LDL-cholesterol >130 mg/dL, HDL-cholesterol <40 mg/dL, or current lipid-lowering treatment. CAD was defined by a documented history of myocardial infarction, angina pectoris, obstructive disease on invasive coronary angiography, or prior coronary revascularization (percutaneous coronary intervention [PCI] or coronary artery bypass grafting [CABG]). Anemia was defined per WHO criteria as hemoglobin <130 g/L in men and <120 g/L in women.

### Data collection and variables

Clinical, demographic, laboratory, and echocardiographic data were systematically extracted from institutional medical records, corresponding to the date of CT imaging. Demographic variables included age, sex, body mass index (BMI), and systolic and diastolic blood pressure. Laboratory assessments encompassed markers of renal function (serum creatinine, serum urea, eGFR), hematological indices (hemoglobin, leukocyte count, neutrophil count, platelet count), systemic inflammation (C-reactive protein, CRP), and metabolic parameters (fasting glucose, total cholesterol, HDL-cholesterol, LDL-cholesterol, triglycerides, uric acid). Echocardiographic data, obtained via transthoracic echocardiography during the same clinical visit, provided measurements of left ventricular ejection fraction (LVEF), LVH, diastolic dysfunction grade, right ventricular systolic pressure (RVSP), and valvular abnormalities (aortic stenosis, mitral regurgitation, tricuspid regurgitation). Aortic stenosis was graded according to the 2014 American Heart Association/American College of Cardiology (AHA/ACC) Valve Guidelines as mild (mean gradient <20 mmHg or aortic valve area [AVA] >1.5 cm^2^), moderate (20–40 mmHg or AVA 1.0–1.5 cm^2^), or severe (>40 mmHg or AVA <1.0 cm^2^). Mitral and tricuspid regurgitation were classified as mild, moderate, or severe based on color-flow and quantitative Doppler criteria, as per European Society of Cardiology (ESC) guidelines. LVH was defined by an LV mass index >115 g/m^2^ in men and >95 g/m^2^ in women. Diastolic dysfunction was graded in accordance with the 2016 American Society of Echocardiography/European Association of Cardiovascular Imaging (ASE/EACVI) recommendations. Valvular abnormalities were analyzed using ``2+'' to indicates moderate or greater severity (grade ≥ 2).

Two distinct secondary imaging outcomes were assessed from the CT acquisition: significant coronary artery stenosis (≥ 50% luminal narrowing) from the CCTA component, and the number of calcified coronary segments from the non-contrast calcium scoring component. These outcomes are reported separately to avoid conflation.

### Coronary CT calcium scoring protocol

Calcium scoring was performed using non-contrast cardiac CT on a Siemens multi-slice CT scanner (Somatom Sensation 4 or 16, Siemens Medical Solutions, Forchheim, Germany) in high-resolution mode. Prospectively electrocardiography (ECG)-triggered axial acquisitions, with a 100 ms exposure, were obtained at 80% of the R-R interval during a single end-inspiratory breath-hold. A total of 40 contiguous 3 mm axial slices were acquired from the tracheal bifurcation to the cardiac apex. Coronary calcifications were identified as hyperdense foci with peak attenuation exceeding 130 Hounsfield units (HU) and spanning a minimum of three contiguous pixels (≥ 1 mm^2^). The Agatston score was computed for each coronary artery (left anterior descending [LAD], left circumflex [LCX], right coronary artery [RCA], and left main [LM]) and summed to yield the total score, based on peak lesion attenuation. Agatston scores were categorized as: 0 (absent calcification), 1–100 (mild), 101–400 (moderate), and >400 (severe) [13]. Image analysis was conducted by experienced cardiovascular radiologists.

**Table 2:** Total Agatston scores and distribution across calcification severity categories in CKD and non-CKD patients

		**CKD group**	**Non-CKD group**	**HL estimate** **[95% CI]**	***P***
Total Agatston scores	Median (IQR)	248.5 (770.3)	198.0 (746.5)	−25.0 [−122.0, 11.0]	0.240^a^
0	*n* (%)	16 (14.5%)	23 (24.0%)	/	
Agatston score categories 1-100	*n* (%)	21 (19.1%)	21 (21.9%)	/	0.056^b^
101–400	*n* (%)	29 (26.4%)	12 (12.5%)	/	
400<	*n* (%)	44 (40.0%)	40 (41.6%)	/	

^a^Mann–Whitney U test, ^b^chi-square test. **Abbreviations**: CI, confidence interval; CKD, chronic kidney disease; HL, Hodges–Lehmann estimator; IQR, interquartile range; *n*, number.

### Ethical statement

This study received approval from the Ethics Committee of the Institute for Cardiovascular Diseases of Vojvodina (approval number: 926-1/2, date of approval: 18.05.2026). As a retrospective study utilizing routinely collected clinical data, the requirement for individual prospective informed consent was waived by the Ethics Committee. Patient data were accessed and analyzed in anonymized form, in strict compliance with institutional data governance procedures. The study was conducted in accordance with the principles outlined in the Declaration of Helsinki.

### Statistical analysis

Statistical analyses were conducted using JASP (version 0.96.0). Continuous variables were assessed for normality using the Shapiro-Wilk test. As the majority exhibited non-normal distributions (Shapiro-Wilk *P* < 0.05), they are reported as median and interquartile range (IQR), with between-group comparisons performed using the Mann-Whitney *U* test. Categorical variables are presented as frequencies and percentages; group differences were evaluated using the chi-squared test or Fisher’s exact test when expected cell counts were fewer than 5. The association between eGFR and Agatston score was quantified using Spearman’s rank correlation, given the non-normal distributions of both variables. Multivariate logistic regression was employed to identify independent predictors of severe coronary calcification (Agatston >400), with candidate variables selected based on clinical relevance. Model performance was assessed using McFadden R^2^, Nagelkerke R^2^, and Tjur R^2^. Results are reported as odds ratios (OR) with 95% confidence intervals (CI). A two-tailed *P* < 0.05 was considered statistically significant throughout the analysis. Baseline demographic and clinical characteristics are summarized in Table 1.

## 2z Results

### Coronary artery calcium scores

Total Agatston scores did not significantly differ between the CKD and control groups (median 248.5 vs. 198.0; Mann-Whitney *U* ═ 4780; *P* ═ 0.240) (Table 2). The distribution of patients across Agatston score categories was also comparable between groups, showing a non-significant trend (chi-squared=7.579; df=3; *P* ═ 0.056), primarily driven by a higher proportion of CKD patients in the 101–400 category (26.4% vs. 12.5%). The proportion with severe calcification (Agatston >400) was 40.0% in the CKD group and 41.6% in controls. The correlation between eGFR and total Agatston score was not statistically significant (Spearman’s *r* = −0.108; *P* ═ 0.123). This correlation was calculated using exact eGFR values, which were available for all CKD patients and for all control patients (eGFR ≥ 60 mL/min/1.73 m^2^).

**Table 3:** Significant coronary artery stenosis ( ≥ 50%) by vessel

		**CKD group**	**Non-CKD group**	**Total**	***P***
LAD	*n* (%)	29 (26.4%)	30 (31.3%)	59 (28.6%)	0.439^b^
LCX	*n* (%)	29 (26.4%)	16 (16.7%)	45 (21.8%)	0.093^b^
RCA	*n* (%)	24 (21.8%)	20 (20.8%)	44 (21.4%)	0.863^b^
LM	*n* (%)	6 (5.5%)	3 (3.1%)	9 (4.4%)	0.415^b^

^b^chi-square test. **Abbreviations**: CKD, chronic kidney disease; LAD, left anterior descending coronary artery; LCX, left circumflex coronary artery; LM, left main coronary artery; N, number; RCA, right coronary artery.

### Individual coronary artery involvement

The following data reflect coronary artery stenosis assessed by CCTA and are distinct from the Agatston calcium score data presented above. No statistically significant difference was observed between CKD and control groups regarding significant stenosis (≥ 50%) in any individual coronary artery: LAD (26.4% vs. 31.3%; *P* ═ 0.439), LCX (26.4% vs. 16.7%; *P* ═ 0.093), RCA (21.8% vs. 20.8%; *P* ═ 0.863), or LM (5.5% vs. 3.1%; *P* ═ 0.415). These vessel-level CCTA findings are summarized in Table 3. Across the entire cohort, significant LAD stenosis was the most common finding (28.6%), followed by LCX (21.8%) and RCA (21.4%). The number of calcified coronary segments was similarly distributed between groups (chi-squared=1.730; df = 4; *P* ═ 0.785).

### Comorbidities and cardiovascular risk factors

Arterial hypertension was significantly more prevalent in the CKD group (90.9% vs. 66.7%; *P* < 0.001; OR = 4.998), as were angina pectoris (30.9% vs. 13.5%; *P* ═ 0.003; OR = 2.858), peripheral arterial occlusive disease (10.0% vs. 2.1%; *P* ═ 0.020; OR = 5.222), and a history of CABG (34.5% vs. 14.6%; *P* ═ 0.001; OR = 3.093). Conversely, prior PCI was less frequent among CKD patients (27.3% vs. 44.8%; *P* ═ 0.009; OR = 0.461). Overall, cardiovascular disease was present in 96.4% of CKD patients vs 68.8% of controls (*P* < 0.001; OR = 12.049). Paradoxically, hyperlipidemia was less frequent in the CKD group (56.4% vs. 74.0%; *P* ═ 0.008; OR = 0.455). No significant differences were found for diabetes mellitus (*P* ═ 0.768), prior myocardial infarction (*P* ═ 0.133), or cerebrovascular disease (*P* ═ 0.640) (Table 4).

**Table 4:** Cardiovascular comorbidities and risk factors in CKD and non-CKD patients

		**CKD group**	**Non-CKD group**	**OR**	***P***
Arterial hypertension	*n* (%)	100 (90.9%)	64 (66.7%)	4.998	<0.001^b^
Diabetes mellitus	*n* (%)	33 (30.0%)	27 (28.1%)	1.095	0.768^b^
Angina pectoris	*n* (%)	34 (30.9%)	13 (13.5%)	2.858	0.003^b^
Myocardial infarction	*n* (%)	33 (30.0%)	20 (20.8%)	1.629	0.133^b^
PAOD	*n* (%)	11 (10.0%)	2 (2.1%)	5.223	0.020^b^
CABG	*n* (%)	38 (34.5%)	14 (14.6%)	3.093	0.001^b^
PCI	*n* (%)	30 (27.3%)	43 (44.8%)	0.461	0.009^b^
Cardiovascular disease	*n* (%)	106 (96.4%)	66 (68.8%)	12.049	<0.001^b^
Hyperlipidemia	*N* (%)	62 (56.4%)	71 (74.0%)	0.455	0.008^b^
Cerebrovascular disease	*n* (%)	10 (9.1%)	7 (7.3%)	1.271	0.640^b^

^b^chi-square test. **Abbreviations**: CABG, coronary artery bypass grafting; CKD, chronic kidney disease; *n*, number; OR, odds ratio; PAOD, peripheral arterial occlusive disease; PCI, percutaneous coronary intervention.

**Figure 1.**
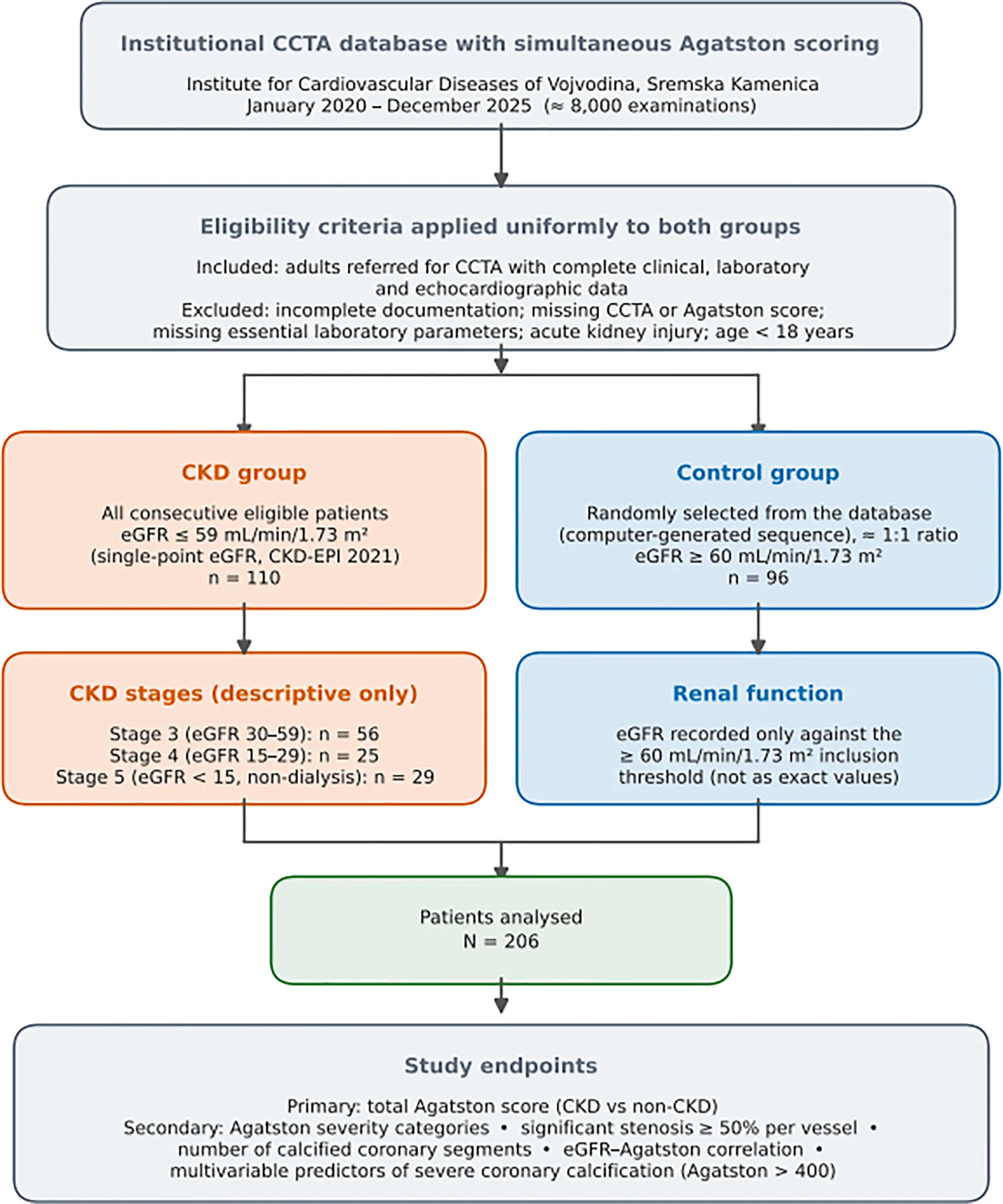
**Study flow and design.** The source population comprised patients undergoing CCTA with simultaneous Agatston scoring at the Institute for Cardiovascular Diseases of Vojvodina between January 2020 and December 2025. Uniform eligibility criteria were applied. The CKD group (eGFR ≤ 59 mL/min/1.73 m^2^, *n* ═ 110) included all consecutive eligible patients. The control group (eGFR ≥ 60 mL/min/1.73 m^2^, *n* ═ 96) was randomly selected to achieve an approximate 1:1 ratio. Due to the non-sequential screening process, a formal screened/excluded patient count and a CONSORT-style flow diagram were not available. **Abbreviations:** CCTA, coronary computed tomography angiography; CKD, chronic kidney disease; CONSORT, Consolidated Standards of Reporting Trials; eGFR, estimated glomerular filtration rate.

**Table 5:** Laboratory parameters in CKD and non-CKD patients

		**CKD group**	**Non-CKD group**	**HL estimate** **[95% CI]**	***P***
Creatinine	Median (IQR)	172.5 (181.5)	81.5 (28.0)	−95.0 [−120.0, −80.0]	<0.001^a^
Urea	Median (IQR)	11.8 (8.6)	6.2 (2.6)	−5.8 [−7.2, −4.8]	<0.001^a^
Uric acid	Median (IQR)	400 (161.8)	322.9 (126.0)	−80.0 [−113.0, −48.0]	<0.001^a^
CRP	Median (IQR)	9.7 (25.5)	3.0 (6.5)	−4.4 [−7.5, −2.6]	<0.001^a^
WBC	Median (IQR)	8.4 (3.5)	8.0 (2.9)	−0.5 [−1.3, 0.19]	0.130^a^
Neu	Median (IQR)	5.9 (2.8)	4.9 (2.6)	−0.98 [−1.58, −0.41]	<0.001^a^
Plt	Median (IQR)	218.5 (96.7)	233.5 (87.0)	6.0 [−14.0, 26.0]	0.563^a^
Hgb	Median (IQR)	121.5 (35.5)	141.5 (19.2)	18.0 [12.0, 24.0]	<0.001^a^
TC	Median (IQR)	2.5 (1.2)	3.5 (1.9)	0.8 [0.46, 1.15]	<0.001^a^
HDL	Median (IQR)	1.1 (0.5)	1.3 (0.4)	0.16 [0.10, 0.24]	<0.001^a^
TG	Median (IQR)	1.4 (0.9)	1.5 (1.2)	0.00003 [−0.2, 0.2]	0.835^a^
Glucose	Median (IQR)	6.8 (3.7)	6.3 (2.0)	−0.3 [−0.8, 0.1]	0.162^a^

^a^Mann-Whitney test. **Abbreviations**: CI, confidence interval; CKD, chronic kidney disease; CRP, C-reactive protein; HDL, high-density lipoprotein cholesterol; Hgb, hemoglobin; HL, Hodges–Lehmann estimator; IQR, interquartile range; Neu, neutrophil count; Plt, platelet count; TC, total cholesterol; TG, triglycerides; WBC, white blood cell count.

### Laboratory parameters

As anticipated, renal function markers were markedly worse in the CKD group: serum creatinine (median 172.5 vs. 81.5 µmol/L; *P* < 0.001) and urea (11.80 vs. 6.20 mmol/L; *P* < 0.001) were significantly elevated. CKD patients also demonstrated significantly higher neutrophil counts, uric acid levels, and CRP, alongside lower hemoglobin, total cholesterol, and HDL-cholesterol (all *P* < 0.001). Fasting glucose, platelet count, total leukocyte count, and triglycerides did not differ significantly between groups (Table 5).

### Echocardiographic parameters

LVEF was significantly lower in the CKD group (median 45.0 vs. 56.5; *P* < 0.001), and RVSP was significantly higher (median 37.0 vs. 28.0 mmHg; *P* < 0.001). Higher-grade valvular pathology was significantly more prevalent in CKD patients: aortic stenosis (*P* ═ 0.048), mitral regurgitation (*P* ═ 0.007), and tricuspid regurgitation (*P* < 0.001). No significant differences were found for LVH (*P* ═ 0.145) or diastolic dysfunction (*P* ═ 0.062) (Table 6). These echocardiographic differences, together with the broader cardiovascular phenotype described above despite comparable CAC burden, are summarized in Figure 2.

**Table 6:** Echocardiographic parameters in CKD and non-CKD patients

		**CKD group**	**Non-CKD group**	**HL estimate [95% CI]**	***P***
LVEF	Median (IQR)	45.0 (23.7)	56.5 (13.2)	9.0 [5.0, 13.0]	<0.001^a^
RVSP	Median (IQR)	37.0 (16.0)	28.0 (13.0)	−8.0 [−11.0, −5.0]	<0.001^a^
AO 2+	*n* (%)	24 (21.8%)	11 (11.5%)	/	0.048^b^
MR 2+	*n* (%)	62 (56.4%)	36 (37.5%)	/	0.007^b^
TR 2+	*n* (%)	53 (48.2%)	20 (20.8%)	/	<0.001^b^
LVH	*n* (%)	53 (48.2%)	56 (58.3%)	/	0.145^b^
DD	*n* (%)	82 (74.5%)	60 (62.5%)	/	0.062^b^

^a^Mann-Whitney test: ^b^chi-Squared Tests; **Abbreviations**: AO, aortic stenosis; CI, confidence interval; CKD, chronic kidney disease; DD, diastolic dysfunction; HL, Hodges–Lehmann estimator; IQR, interquartile range; LVEF, left ventricular ejection fraction; LVH, left ventricular hypertrophy; MR, mitral regurgitation; *n*, number; RVSP, right ventricular systolic pressure; TR, tricuspid regurgitation.

**Figure 2.**
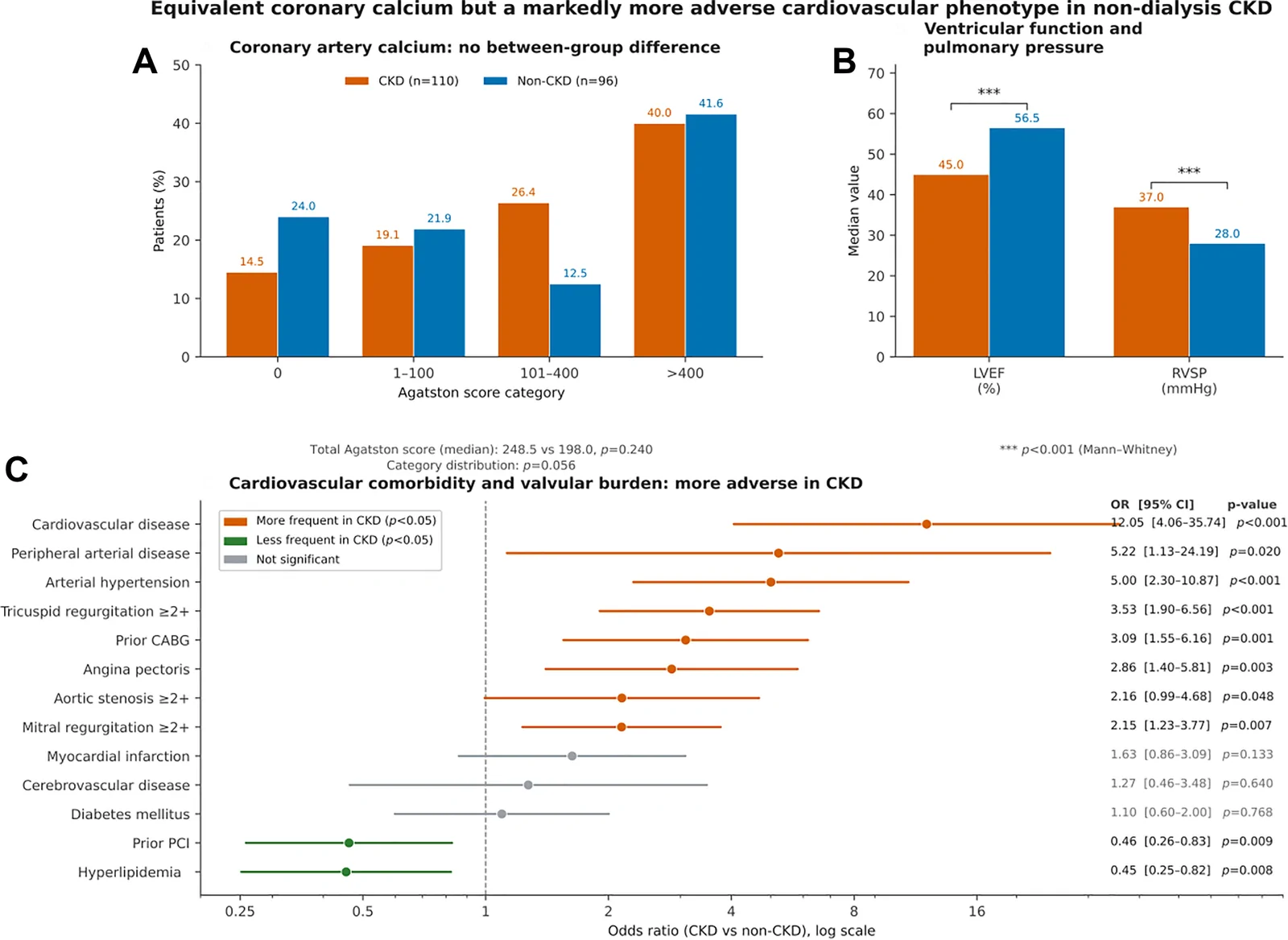
**Equivalent coronary artery calcium burden but a markedly more adverse cardiovascular phenotype in non-dialysis CKD.** (A) The distribution of patients across Agatston score categories did not differ significantly between groups (*P ═* 0.056); total Agatston score was also comparable (median 248.5 vs 198.0; *P ═* 0.240). (B) LVEF was significantly lower and RVSP was significantly higher in the CKD group (both *P* < 0.001, Mann–Whitney *U* test). (C) Forest plot of odds ratios (CKD vs non-CKD) for cardiovascular comorbidities and higher-grade valvular disease; odds ratios and 95% confidence intervals were derived from the contingency-table counts. Orange denotes conditions more frequent in CKD (*P <* 0.05), green denotes conditions less frequent in CKD (*P* < 0.05), and grey denotes non-significant associations. The dashed line marks an odds ratio of 1. **Abbreviations:** CABG, coronary artery bypass grafting; CI, confidence interval; CKD, chronic kidney disease; LVEF, left ventricular ejection fraction; n, number of patients; OR, odds ratio; PCI, percutaneous coronary intervention; RVSP, right ventricular systolic pressure.

### Predictors of severe coronary calcification

In the prespecified multivariable logistic regression model, which included CKD status, diabetes mellitus, arterial hypertension, prior CABG, age, and sex, CKD status was not independently associated with severe coronary calcification (adjusted OR 0.84, *P* ═ 0.576). No covariate achieved statistical significance, although age demonstrated a borderline association (adjusted OR 1.03 per year, *P* ═ 0.051). Overall, the model exhibited modest performance (Table 7). The regression model is presented as associative; no causal inference regarding individual covariates is intended, and the covariate set was prespecified prior to analysis.

## Discussion

The principal finding of this study is that the total coronary artery calcium burden, quantified by the Agatston score, did not differ significantly between patients with CKD stages 3–5 and controls with preserved renal function recruited from the same cardiovascular referral population (median 248.5 vs. 198.0; *P* ═ 0.240). Furthermore, after controlling for traditional cardiovascular risk factors, CKD status did not emerge as an independent predictor of severe calcification.

Notably, the distribution across Agatston categories showed a borderline non-significant trend (*P* ═ 0.056), primarily driven by a higher proportion of CKD patients in the moderate calcification range (101–400: 26.4% vs. 12.5%). This pattern may reflect the early-to-intermediate stage of CKD-related vascular remodeling in this non-dialysis cohort, where medial calcification begins to accumulate without yet reaching the extreme calcium loads observed in dialysis patients. Given the small sample size and borderline *P*-value, this observation should be regarded as hypothesis-generating rather than conclusive.

The absence of a significant eGFR–Agatston correlation (Spearman’s r=–0.108; *P* ═ 0.123) further supports this interpretation. Even when eGFR is treated as a continuous variable rather than a binary classifier, the degree of residual renal function does not predict calcium burden within this high-risk referral population. This correlation was calculated using exact eGFR values available for all patients in both groups.

This finding appears to contradict earlier reports of higher CACS in CKD, but those studies predominantly compared CKD patients with lower-risk general population or outpatient controls [6, 9, 10]. When both groups share the same high-risk referral setting, albeit with substantially different comorbidity burdens, as in the present study, the incremental contribution of reduced GFR to coronary calcium burden is attenuated or eliminated. Data from the Chronic Renal Insufficiency Cohort (CRIC) Study and the Korean Cohort Study for Outcome in Patients With Chronic Kidney Disease (KNOW-CKD) cohort corroborate this interpretation: after adjustment for traditional cardiovascular risk factors, the independent contribution of CKD-specific determinants to cross-sectional coronary calcium burden varied substantially across reference populations [9, 10]. The CRIC Study does, however, demonstrate the clear prognostic relevance of longitudinal coronary artery calcium (CAC) progression within a CKD cohort—incident CAC conferred a 2.42-fold increased risk of atherosclerotic events, and progression ≥ 50 Agatston units per year was associated with a 1.73-fold increased mortality risk [14]—underscoring that cross-sectional burden and longitudinal trajectory carry distinct information.

The mechanistic interpretation that follows is grounded in established pathophysiology from the literature rather than directly measured variables in this cohort. Since phosphate, PTH, FGF-23, and other CKD-Mineral and Bone Disorder (MBD) parameters were not available in this dataset, the following discussion should be read as contextualization rather than as mechanistic inference from our data.

The plausible mechanistic explanation for equivalent total calcium scores despite markedly different clinical profiles lies in the dual nature of vascular calcification in CKD. In the general population, calcification is predominantly intimal, arising within atherosclerotic plaques as a consequence of lipid-driven inflammation and macrophage activity, directly reflecting obstructive disease burden. In CKD, a second pathway—medial sclerosis driven by hyperphosphatemia, elevated FGF-23, reduced Klotho expression, and secondary hyperparathyroidism—becomes progressively dominant as renal function declines [3, 4, 7]. Medial calcification occurs in vessels with minimal atherosclerotic burden, producing a diffuse circumferential pattern not confined to areas of plaque [7]. The Agatston method captures both intimal and medial calcium indiscriminately and cannot differentiate between them [13]; total calcium mass may therefore be equivalent across groups while the underlying pathological substrate diverges fundamentally. Intimal and medial calcification carry distinct vascular consequences: intimal calcium reflects atheromatous plaque and stenotic risk, whereas medial calcium produces arterial stiffness, systolic hypertension, and increased ventricular afterload without necessarily narrowing the lumen [15, 16]. This mechanistic divergence implies that equivalent Agatston scores in CKD and non-CKD patients may represent qualitatively different and prognostically discordant processes. Coronary calcification in CKD is thus a heterogeneous process, and a single unidimensional score cannot capture this complexity [15].

**Table 7:** Multivariable logistic regression for severe coronary artery calcification (Agatston score >400)

	**Adjusted OR**	**95% CI**	***P***
CKD status	0.84	0.45–1.56	0.576
Diabetes mellitus	0.65	0.34–1.27	0.195
Arterial hypertension	1.49	0.70–3.16	0.301
Prior CABG	0.60	0.30–1.20	0.148
Age (per 1 year)	1.03	1.00–1.06	0.051
Male sex	1.07	0.59–1.94	0.830

Dependent variable: severe coronary calcification, defined as an Agatston score >400. The overall model performance was modest (McFadden R^2^ ═ 0.029; Nagelkerke R^2^ ═ 0.052). **Abbreviations**: CABG, coronary artery bypass grafting; CI, confidence interval; CKD, chronic kidney disease; OR, odds ratio; R^2^, pseudo-coefficient of determination.

The clinical consequences of medial calcification predominance on CAC score interpretation have been directly quantified by Jansz et al. in a propensity score-matched cohort of 112 dialysis patients and 112 non-CKD controls undergoing coronary CT angiography. Despite 3.6-fold higher median Agatston scores in dialysis patients, the prevalence of significant stenosis (≥ 50%) was virtually identical between groups (35% vs. 36%), and each 100-unit CAC increment conferred approximately 1.5-fold lower odds of stenosis in dialysis patients than in non-CKD patients (OR 0.67; 95% CI 0.52–0.83) [17]. Even among dialysis patients with CAC >400, only 20% had ≥ 70% stenosis, compared with 43% in non-CKD patients [17]. These findings directly challenge the applicability of standard CAC risk thresholds in the CKD population, including the ≥ 400 HU threshold used in the present study. It should be noted, however, that the Jansz cohort comprised patients on chronic dialysis, and these findings may not translate directly to the non-dialysis CKD population studied here. An additional statistical ceiling effect further limited between-group discrimination in our cohort: over 40% of patients in both groups exceeded Agatston >400, compressing the discriminatory range of the score. The score’s additional recognized limitations—inability to account for coronary distribution, calcium density, or left main artery involvement—further reduce its utility as a risk stratification tool in uniformly high-risk populations [18, 19]. High total CACS does not reliably identify culprit plaque vulnerability at the lesion level [20–22].

Despite equivalent Agatston scores, CKD patients exhibited a substantially more adverse multidimensional cardiovascular phenotype, confirming that coronary calcium burden alone does not capture the full extent of CKD-related cardiovascular disease. The older median age of CKD patients compared to controls is an expected finding in a CKD cohort and reflects the progressive accumulation of renal injury with age. Importantly, age was included as a covariate in the multivariable logistic regression model, where it demonstrated only a borderline association with severe calcification (adjusted OR 1.03 per year, *P* ═ 0.051), suggesting that the age difference between groups does not fully explain the equivalent Agatston scores and that residual age-related confounding, while possible, is unlikely to be the dominant driver of the observed findings.

Arterial hypertension was present in 90.9% vs. 66.7% of controls (*P* < 0.001; OR = 4.998), driven by sodium and fluid retention, renin-angiotensin-aldosterone activation, and direct vascular stiffening from medial calcification [2, 5]. Angina pectoris was twice as prevalent (30.9% vs. 13.5%; *P* ═ 0.003), and peripheral arterial occlusive disease five times more frequent (10.0% vs. 2.1%; *P* ═ 0.020), reflecting the diffuse multi-territory atherosclerosis characteristic of CKD, driven by systemic inflammation, oxidative stress, and uremic toxin-mediated endothelial injury [1]. The substantially higher rate of prior CABG (34.5% vs. 14.6%; *P* ═ 0.001; OR = 3.093) and conversely lower PCI rate (27.3% vs. 44.8%; *P* ═ 0.009) in the CKD group reflect greater anatomical complexity and the evidence-based preference for surgical revascularization in CKD patients with multi-vessel calcified disease, where percutaneous outcomes are suboptimal, and the well-recognized tendency to avoid nephrotoxic iodinated contrast media in patients with already compromised renal function, which further reduces the uptake of PCI in this population [1, 6, 23]. In the revised adjusted model, prior CABG was not independently associated with severe calcification, underscoring the need for cautious, non-causal interpretation of the severe CAC analysis.

Echocardiographic findings confirmed structural cardiac remodeling extending well beyond CAD. Median LVEF was significantly lower in CKD patients (45.0% vs. 56.5%; *P* < 0.001), and RVSP markedly elevated (37.0 vs. 28.0 mmHg; *P* < 0.001), consistent with the established entity of CKD-associated cardiomyopathy—a condition driven by pressure overload from hypertension, volume overload from fluid retention and anemia, and direct myocardial injury mediated by uremic toxins, FGF-23 excess, and chronic sympathetic activation causing interstitial fibrosis [2, 5]. More advanced grades of aortic stenosis (*P* ═ 0.048), mitral regurgitation (*P* ═ 0.007), and tricuspid regurgitation (*P* < 0.001) in the CKD group reflect accelerated valvular calcification from disturbed mineral metabolism and hemodynamic stress from chronic volume overload [3, 5]. These structural abnormalities are entirely invisible to CACS and emphasize the necessity of systematic echocardiographic evaluation in this population.

The paradoxically lower hyperlipidemia prevalence (56.4% vs. 74.0%; *P* ═ 0.008) and significantly reduced total and HDL-cholesterol in the CKD group reflect the malnutrition-inflammation-atherosclerosis (MIA) syndrome and the well-recognized phenomenon of reverse epidemiology in advanced CKD [24]. Diabetes mellitus was present with equal prevalence across groups (*P* ═ 0.768) and did not modify calcification severity in this cohort, consistent with the observation that the CKD-related calcification burden in this referral population was driven primarily by shared cardiovascular comorbidities rather than glycemic status.

These findings have two main clinical implications for cardiovascular risk stratification of CKD patients in cardiology referral settings. First, a high Agatston score in a CKD patient should not be reflexively attributed to kidney disease when shared cardiovascular risk factors are present; equally, a low or absent CACS does not exclude the profoundly adverse cardiovascular phenotype demonstrated in this cohort. Standard CAC risk thresholds derived from general population studies cannot be directly applied in CKD without accounting for the contribution of medial calcification, which may inflate the Agatston score without a proportionate increase in obstructive coronary disease. Second, and most importantly, the cardiovascular burden of CKD is multidimensional and cannot be adequately captured by CACS alone; systematic integration of echocardiography, systemic vascular evaluation, and detailed revascularization history is essential for comprehensive risk assessment in this population. These conclusions are hypothesis-generating and require prospective validation in larger, ideally multicenter cohorts before any modification of clinical risk stratification practice could be considered.

### Limitations

Several limitations warrant consideration. The retrospective, single-center design precludes causal inference and may introduce selection bias inherent to clinical registry data, potentially limiting the generalizability of results to CKD patients managed exclusively in nephrology settings or those with less advanced cardiovascular disease. CKD staging relied on a single creatinine measurement rather than the longitudinal confirmation required by KDIGO criteria, which could lead to misclassification, particularly for patients with borderline eGFR values (50–65 mL/min/1.73 m^2^). Furthermore, the CKD group exhibited biological heterogeneity, spanning eGFR values from <15 to 59 mL/min/1.73 m^2^. Stage-specific analyses of Agatston scores across CKD stages 3, 4, and 5 were not performed due to insufficient sample sizes within individual strata to support stable subgroup comparisons. For descriptive purposes, the CKD group comprised 56 patients with stage 3 (eGFR 30–59 mL/min/1.73 m^2^), 25 with stage 4 (eGFR 15–29 mL/min/1.73 m^2^), and 29 with stage 5 (eGFR <15 mL/min/1.73 m^2^). Therefore, the binary eGFR threshold should be regarded as a pragmatic classification rather than a mechanistically precise stratification. Future studies with larger CKD cohorts should examine whether calcification burden differs across advancing stages of renal dysfunction.

Individual eGFR values for the control group were not systematically recorded beyond the inclusion threshold of ≥ 60 mL/min/1.73 m^2^, precluding a full distributional comparison of eGFR across groups. This represents a methodological limitation; future studies should document the complete eGFR spectrum in both groups for more granular renal function characterization. This constitutes a central validity constraint, and all conclusions regarding ‘CKD stages 3–5’ should be interpreted as referring to an eGFR-defined stratum rather than formally confirmed CKD. A sensitivity analysis excluding patients with eGFR 50–65 mL/min/1.73 m^2^ was not performed due to sample size constraints but would be warranted in a larger cohort to confirm the robustness of the primary finding [11].

Key CKD-MBD determinants of vascular calcification—including serum phosphate, parathyroid hormone, FGF-23, dialysis vintage, and use of phosphate binders or calcimimetics—were not systematically available, precluding adjustment for these variables in the regression models. This represents a fundamental disconnect between the biological narrative presented in the Introduction and Discussion and the variables actually measured, thereby limiting the mechanistic conclusions that can be drawn from this dataset.

Formal propensity score matching for cardiovascular confounders was not performed, thus residual confounding cannot be excluded. In particular, substantial baseline imbalances between groups—including higher rates of arterial hypertension (90.9% vs. 66.7%), prior coronary artery bypass grafting (CABG) (34.5% vs. 14.6%), and markedly lower LVEF (45.0 vs. 56.5%) in the CKD group—represent sources of residual confounding that the adjusted model cannot fully account for, given the available sample size and the absence of propensity-based matching.

Dichotomizing the Agatston score at >400 introduced a ceiling effect, as approximately 40% of patients in both groups exceeded this threshold, reducing statistical power to detect between-group differences. No ordinal logistic regression sensitivity analysis was performed; the binary logistic regression presented in Table 7 represents the sole regression analysis conducted in this study. Ordinal logistic regression across the four established Agatston categories is identified as a priority for future larger-cohort studies where adequate statistical power would allow stable model estimation.

Furthermore, given the substantial differences in prior revascularization history between groups—particularly the higher prevalence of prior CABG in CKD patients (34.5% vs. 14.6%)—stratified analyses and formal interaction testing (CKD status × prior CABG) would have been informative. However, the available sample size (*n* ═ 206 with 84 events) is insufficient to support stable interaction testing, which typically requires four times the sample size of the main analysis. These analyses are therefore identified as a priority for future larger-cohort studies.

A formal validated cardiovascular risk score (such as the ESC SCORE2 or Framingham Risk Score) was not incorporated into the study design or adjusted analyses. In this uniformly high-risk referral population—where established CAD was present in 96.4% of CKD and 68.8% of non-CKD patients—global risk score application would have limited discriminatory value. Nevertheless, the absence of a standardized baseline risk framework is acknowledged as a limitation. Similarly, coronary disease severity was characterized by vessel-level stenosis and revascularization history rather than a structured CAD scoring system (e.g., SYNTAX score), which limits the depth of coronary burden assessment.

Finally, the cross-sectional design captures only a single time-point calcium measurement. Longitudinal studies with serial CACS assessments, advanced plaque characterization, and mineral metabolism profiling are needed to determine whether the equivalent calcium burden observed here persists over time and whether its prognostic significance differs between CKD and non-CKD patients in high-risk referral settings.

## Conclusion

In this cardiovascular referral cohort, patients with eGFR ≤ 59 mL/min/1.73 m^2^, classified as CKD stages 3–5 based on single-point eGFR estimation, were not independently associated with a greater coronary artery calcium burden compared to patients with preserved renal function drawn from the same high-risk referral population. Total Agatston score, categorical severity distribution, individual vessel involvement, and the eGFR–Agatston correlation were all non-significant, suggesting that, in this selected referral cohort, an independent association between eGFR-defined renal impairment and greater CACS burden was not demonstrated beyond shared cardiovascular comorbidities. Nevertheless, CKD patients demonstrated a substantially more adverse cardiovascular phenotype—encompassing impaired ventricular function, elevated pulmonary pressures, and advanced valvular disease. These findings emphasize that the cardiovascular burden of CKD is multidimensional and cannot be adequately captured by calcium scoring alone. Systematic multiparametric cardiac assessment, integrating echocardiography and systemic vascular evaluation alongside CACS, is essential for comprehensive risk stratification in this high-risk population.

## Data Availability

The data supporting the findings of this study are not publicly available due to patient privacy and confidentiality restrictions, but are available from the corresponding author upon reasonable request.
